# Safety and neurochemical profiles of acute and sub-chronic oral treatment with agmatine sulfate

**DOI:** 10.1038/s41598-019-49078-0

**Published:** 2019-09-03

**Authors:** David H. Bergin, Yu Jing, Gail Williams, Bruce G. Mockett, Hu Zhang, Wickliffe C. Abraham, Ping Liu

**Affiliations:** 1 Department of Anatomy, School of Biomedical Sciences, Brain Health Research Centre, Brain Research New Zealand, University of Otago, Dunedin, New Zealand; 2 School of Pharmacy, Brain Health Research Centre, Brain Research New Zealand, University of Otago, Dunedin, New Zealand; 3 Department of Pathology, University of Otago, Dunedin, New Zealand; 4 Department of Psychology, Brain Health Research Centre, Brain Research New Zealand, University of Otago, Dunedin, New Zealand

**Keywords:** Molecular neuroscience, Molecular biology

## Abstract

Agmatine (decarboxylated arginine) exerts numerous central nervous system (CNS) dependent pharmacological effects and may potentially modulate altered neurochemistry seen in neurological disorders. In preclinical studies, injection has been the predominant route of systemic administration. However, a significant translational step would be the use of oral agmatine treatment at therapeutic doses and better understanding of L-arginine metabolic profiles in the CNS post-treatment. The present study systematically investigated the tolerability, safety and brain-plasma neurochemistry following daily oral agmatine sulfate treatment (via gavage) to wild-type (WT) mice up to 900 mg/kg for one week (Experiment 1) or WT and APPswe/PS1ΔE9 transgenic (Tg) mice at 300 mg/kg for fifteen weeks (Experiment 2). Agmatine treatment in both experiments was well tolerated with no marked behavioural impairments, and gross necropsy and organ histology revealed no pathological alterations after 15-week dosing. Moreover, oral treatment increased agmatine levels in the hippocampus and plasma of WT mice (Experiment 1), and in 6 brain regions examined (but not plasma) of WT and Tg mice (Experiment 2), at 30 minutes or 24 hours post-treatment respectively. This study provides fundamental pre-clinical evidence that daily oral delivery of agmatine sulfate to both WT and Tg mice is safe and well tolerated. Exogenous agmatine passes through the blood brain barrier and accumulates in the brain to a greater extent in Tg mice. Furthermore exogenous agmatine has differential actions in the brain and periphery, and its effect on brain putrescine appears to be dependent on the time post-treatment.

## Introduction

Agmatine (decarboxylated arginine) is widely and unevenly distributed in the mammalian brain as a neuromodulator^1,2^, and may directly participate in learning and memory processes^3–6^. Agmatine also modulates the balance between other L-arginine metabolic pathways via its influence on the production of nitric oxide (NO) and polyamines putrescine, spermidine and spermine^7–9^. Furthermore, abundant pre-clinical evidence indicates that systemic treatment with agmatine sulfate, the commonly used salt form of agmatine (and herein termed agmatine for the exogenous substance), is ‘capable of synergistically modulating, directly or indirectly, multiple dysregulated molecular targets to ameliorate various complex clinical disorders’ when given to rodents (predominantly by injectable routes of administration), hence agmatine being referred to as a ‘magic shotgun’ (reviewed in^8,10,11^). However daily injection of agmatine limits its clinical translation. Under conditions of oral delivery and subcutaneous injection, agmatine has similar anxiolytic and anti-depressant effects when rats were assessed in the light/dark transition test, Vogel’s conflict drinking and social interaction tasks^12^. More recently, oral agmatine has been shown to be more potent in behavioural models of stress and depression than conventional anti-depressants, and potentiates their effects when co-administered at sub-effective doses via its interactions with N-methyl-D-aspartate (NMDA) receptors and the arginine-NO pathway^13–17^, illustrating its wide neuromodulatory mechanisms of action.

As a therapy, agmatine treatment is considered relatively safe. No toxicity or adverse effects were reported in the positional sense and gait tests for up to 6 hours following a single oral dose up to 480 mg/kg in rats^18^. For longer term treatment, agmatine has been administered daily to rats via the drinking water at 100 mg/kg for 95 days with no obvious negative effects on behaviour or signs of toxicity, suggesting the safety of sub-chronic dietary agmatine^19^. Unfortunately the delivery of agmatine via drinking water would require high concentrations in the water in order to achieve a therapeutic range in the brain, due to the small quantities consumed at one time, limited blood brain barrier (BBB) permeability and agmatine’s rapid clearance from the blood via urine^20,21^. For a number of years, agmatine has also been taken as nutraceutical dietary supplement albeit at a lower 37–46 mg/kg dosage in a case study by the Gilads, who provided detailed blood and urine analyses^22^. However, it is unclear how brain neurochemistry changes following agmatine treatment.

Following safety, efficacy is paramount in preclinical studies, especially when tested as a therapeutic in pathological conditions. We have previously reported that intraperitoneal (IP) injection of agmatine protects against behavioural deficits in the injectable amyloid-beta (Aβ_25–35_) model of Alzheimer’s disease (AD)^23^, and that L-arginine metabolism in the brain is altered following the intracerebroventricular infusion of pre-aggregated Aβ_25–35_ and in APPswe/PS1ΔE9 mice with chronic amyloid accumulation and in patients with AD^24–27^. It is of interest to note that APPswe/PS1ΔE9 mice at 13 (but not 7) months of age display behavioural deficits in spatial water maze tasks and altered arginine metabolic profiles in the brain, such as reduced agmatine levels in the hippocampus and increased amino acid and polyamine levels in multiple regions, demonstrating the parallel development of altered brain arginine metabolism and behavioural deficits in this mouse model of AD^26^. These preclinical studies complement findings in post-mortem human brain tissue from AD patients^27^, implicating altered arginine metabolism in AD neuropathogenesis and cognitive impairment. The amyloid centric models are therefore ideal for testing the efficacy of oral agmatine treatment.

**Figure 1:**
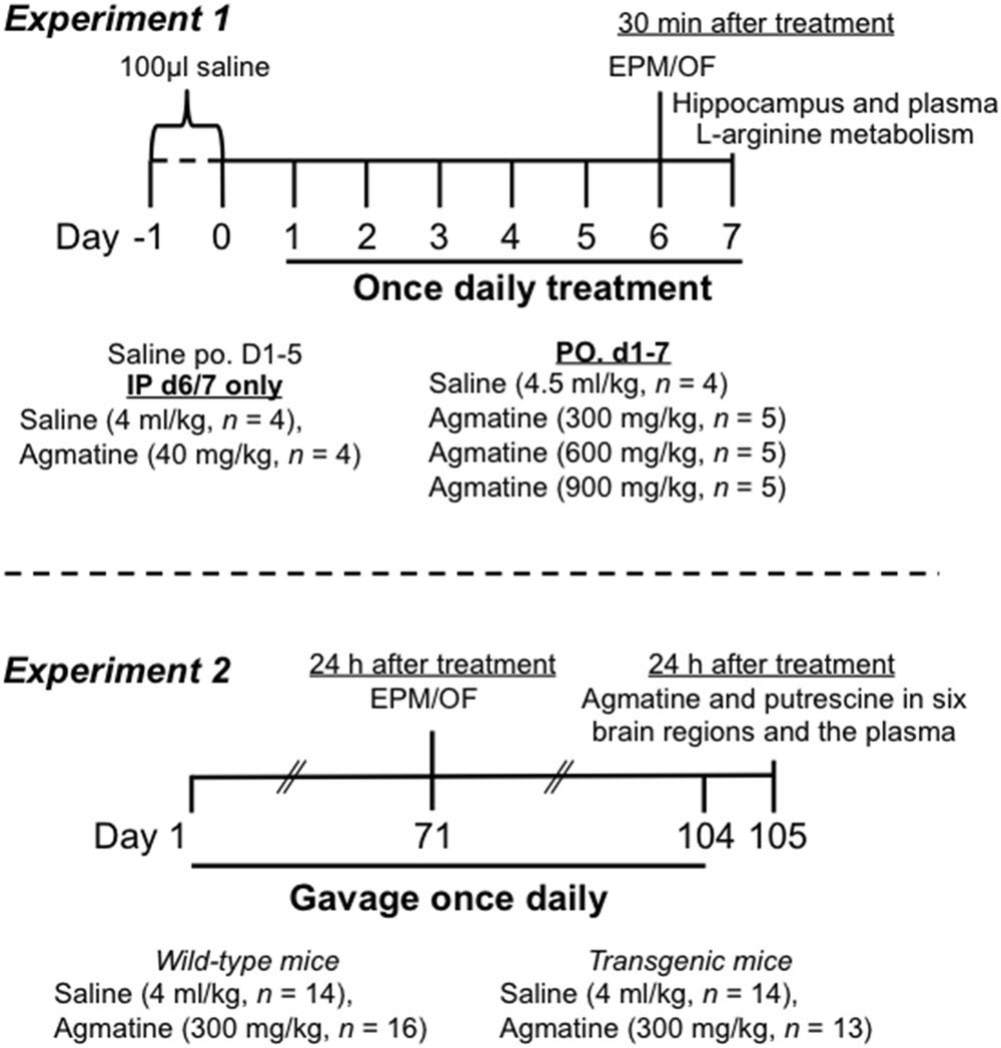
Experimental timelines of short-term (Experiment 1) and sub-chronic (Experiment 2) agmatine treatment. In Experiment 1, wild-type mice were gavaged with 100 μl saline once daily for two days. During the following 7 days, animals were given saline gavage on days 1–5 but intraperitoneal (IP) injection of saline or agmatine at 40 mg/kg once daily on days 6 and 7, whereas others received daily oral gavage of saline or agmatine at 300, 600 and 900 mg/kg. On day 6, all animals were tested in the elevated plus maze (EPM) and open field (OF) 30 minutes post-treatment. On day 7, animals were sacrificed 30 minutes post-treatment followed by blood (plasma) and brain tissue (hippocampus) collection for quantification of L-arginine and its metabolites. In Experiment 2, wild-type and APP/PS1 transgenic mice received oral gavage of saline or agmatine sulfate at 300 mg/kg once daily for 5 days per week for 8 weeks, then once daily for 7 days a week for a further 7 weeks. On day 71, animals were tested in the EPM and OF 24 h after treatment. On day 105, animals were sacrificed 24 hours after the final treatment of day 104 followed by blood and brain tissue collection for agmatine and putrescine quantification.

The present study was designed to determine the tolerability, safety and brain and plasma arginine metabolic profiles in wild-type and APPswe/PS1ΔE9 (Tg) mice following daily agmatine treatment by oral gavage. Experiment 1, termed the short-term dose-ranging agmatine treatment study, was a dose-response pilot study. The behavioural effects of agmatine sulfate at 0, 300, 600 and 900 mg/kg once daily for one week were examined using the elevated plus maze and open field apparatus 30 min post-gavage (Fig. 1). To ascertain agmatine transport across the BBB, the agmatine levels in the brain tissue (hippocampus) and plasma were measured at the time point of 30 min after the final gavage. Moreover, the effects of exogenous agmatine on arginine metabolic profiles in the brain and blood were determined, by measuring the levels of L-arginine and its downstream metabolites (L-citrulline, L-ornithine, glutamate, glutamine, γ-aminobutyric acid (GABA), putrescine, spermidine and spermine) in the hippocampus and plasma at the same time point. These findings were compared with the behaviourally and neurochemically effective IP dose of agmatine at 40 mg/kg^23,28–30^, injected once daily for the final two days. The matched IP-oral dose of 300 mg/kg po was then used in Experiment 2, termed the sub-chronic agmatine treatment study. WT and Tg mice were treated once daily for 15 weeks followed by the behavioural tests and the measurements of agmatine and putrescine levels in the brain and plasma 24 h after the final treatment (Fig. 1). Because exogenous agmatine accumulates in the organs where it exerts beneficial effects^8,20,21,31^, gross necropsy and major organs were subject to histological examinations at the conclusion of Experiment 2 to further assess the safety of the sub-chronic oral treatment of agmatine.

## Results

### Experiment 1: Short-term dose-ranging agmatine treatment

All animals that entered Experiment 1 completed the study without health complications, indicating that daily oral agmatine treatment at doses up to 900 mg/kg for one week was tolerable. When body weights were compared between the first and final days of treatment, there was a significant day effect (*F*(1,16) = 11.09, *P* = 0.004), with all animals losing weight over the course of the study (Table 1), as the first week of gavaging was stressful to all animals. There was however no significant effect of treatment or treatment × day interaction.

**Table 1:** Effects of short-term agmatine treatment on body weight.

Body weight (g)	Agm-0 (IP)	Agm-40 (IP)	Saline (oral)	Agm-300 (oral)	Agm-600 (oral)	Agm-900 (oral)
Day 1	40.20 ± 3.37	38.05 ± 3.55	41.12 ± 2.7	41.86 ± 2.55	41.42 ± 1.99	42.06 ± 2.72
Day 7	39.9 ± 3.38	37.93 ± 3.05	39.75 ± 2.49	41.08 ± 2.06	40.7 ± 2.53	41.46 ± 2.69

Mean (± SEM) body weight on days 1 and 7 for animals receiving intraperitoneal (IP) injection of agmatine at 0 (Agm-0) or 40 (Agm-40) mg/kg, and oral administration (oral) of agmatine at 0 (Agm-0), 300 (Agm-300), 600 (Agm-600) or 900 (Agm-900) mg/kg (n = 4–5/group).

#### Behavioural results

**Figure 2:**
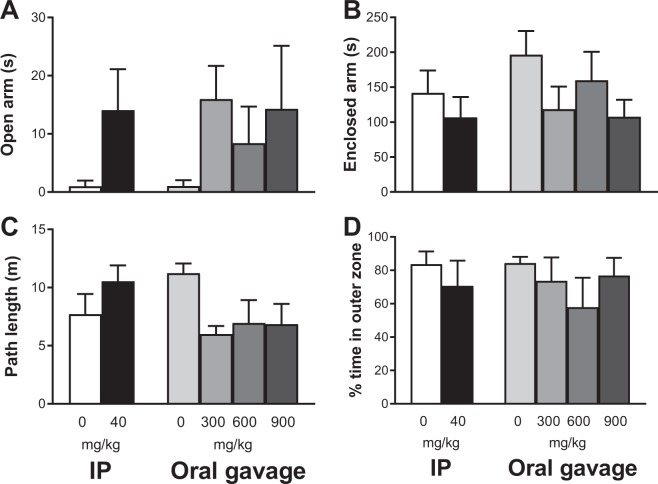
Behavioural effects of short-term agmatine treatment. Mean (±SEM) time spent in open (**A**) and enclosed (**B**) arms of the elevated plus maze, and path length (**C**) and % time spent in the outer zone (**D**) of the open field apparatus in wild-type mice received intraperitoneal (IP) injection of agmatine at 0 or 40 mg/kg or oral gavage of agmatine at 0, 300, 600 and 900 mg/kg (n = 4–5/group).

Agmatine treatment by IP injection or oral gavage had no effects on animals’ behaviour in the elevated plus maze in terms of arm preference, based on the duration spent in open and closed arms (Fig. 2A,B) and the total number of entries to open and closed arms (data not shown). Moreover, both IP injection and oral gavage of agmatine had no significant effects on the path length travelled or the percentage of time in the outer zone during the open field test (Fig. 2C,D). However, there was a trend (*P* = 0.056) of decreased path length in agmatine treated mice at 300 mg/kg when compared to the saline treated controls.

#### Neurochemical results

**Figure 3:**
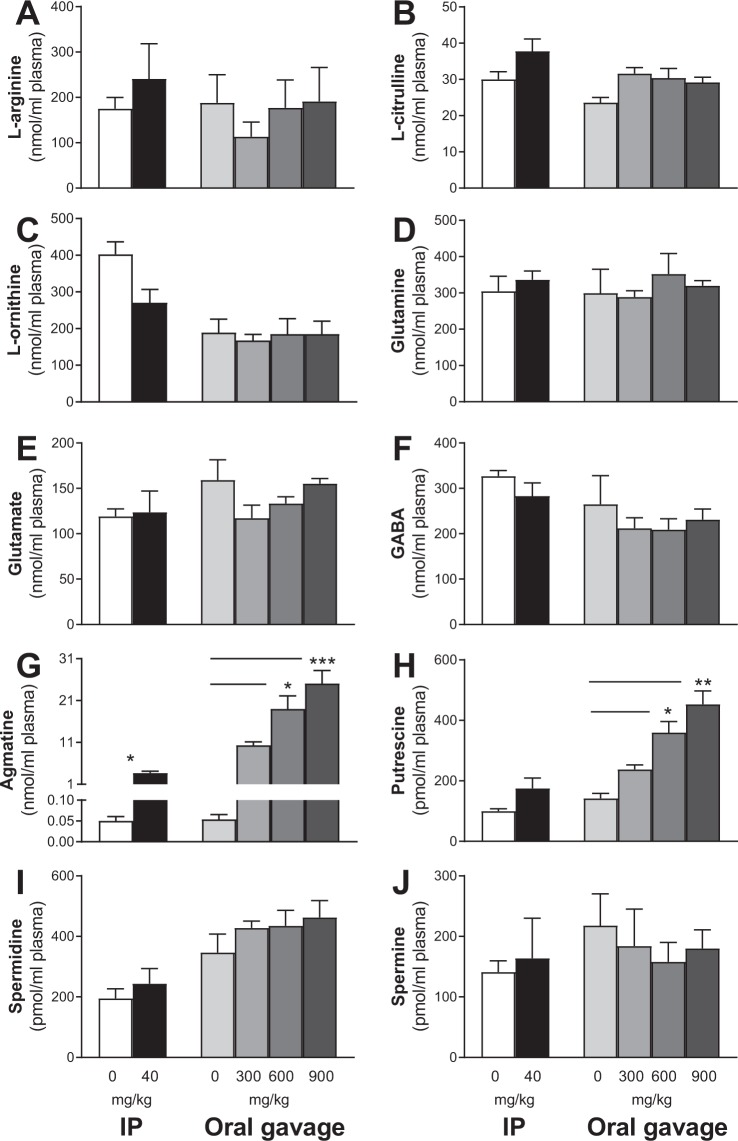
Short-term agmatine treatment effects on plasma L-arginine metabolism. Mean (±SEM) L-arginine (**A**), L-citrulline (**B**), L-ornithine (**C**), glutamine (**D**), glutamate (**E**), GABA (**F**), agmatine (**G**), putrescine (**H**), spermidine (**I**) and spermine (**J**) concentrations in the plasma of wild-type mice 30 min post-treatment with agmatine sulfate by intraperitoneal (IP) injection at 0 or 40 mg/kg or oral gavage at 0, 300, 600 and 900 mg/kg (n = 4–5/group). Treatment resulted in dose-dependent increases in plasma agmatine and putrescine levels, mainly in the oral treatment groups. *Indicates significant treatment effects compared with the saline group at *p < 0.05, **p < 0.01 or ***p < 0.001.

All animals were sacrificed 30 min after the final oral gavage or IP injection. Trunk blood and the whole hippocampus were collected from each animal to determine plasma and tissue concentrations of L-arginine and its down-stream metabolites (including agmatine). Regarding the plasma profile, there were no significant differences between groups in L-arginine (Fig. 3A), L-citrulline (Fig. 3B), L-ornithine (Fig. 3C), glutamine (Fig. 3D), glutamate (Fig. 3E), GABA (Fig. 3F), spermidine (Fig. 3I) and spermine (Fig. 3J) regardless of the delivery route and dose of agmatine. Injection of agmatine at 40 mg/kg resulted in increased agmatine level in plasma (*P* = 0.03, Fig. 3G). For mice treated orally with agmatine at 0 (saline-treated), 300, 600 and 900 mg/kg, one-way ANOVA revealed significant dose-dependent increases in plasma agmatine (*P* = 0.0012, Fig. 3G) and putrescine (*P* = 0.0012, Fig. 3H). Post-hoc tests revealed higher agmatine and putrescine concentrations in mice treated with agmatine at the doses of 600 (*P* < 0.05) and 900 (*P* < 0.001) mg/kg when compared to saline treated mice.

**Figure 4:**
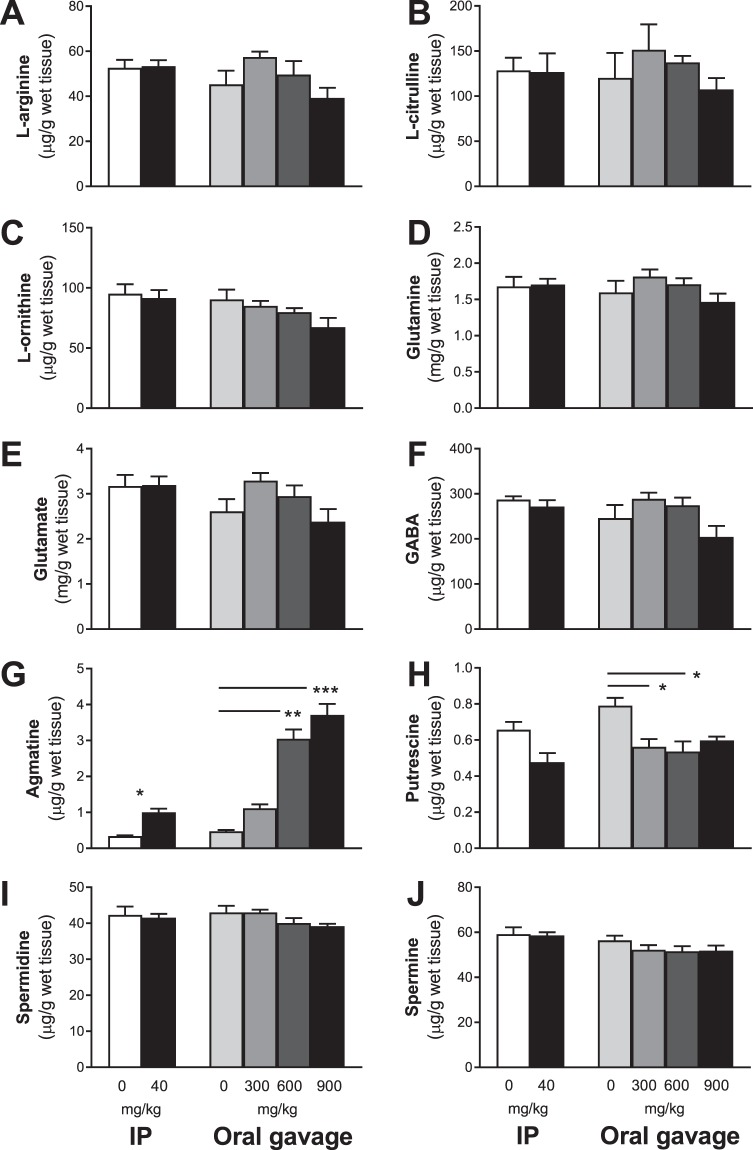
Short-term agmatine treatment effects on hippocampal L-arginine metabolism. Mean (±SEM) L-arginine (**A**), L-citrulline (**B**), L-ornithine (**C**), glutamine (**D**), glutamate (**E**), GABA (**F**), agmatine (**G**), putrescine (**H**), spermidine (**I**) and spermine (**J**) concentrations in the whole hippocampus of wild-type mice 30 min post-treatment with agmatine sulfate by intraperitoneal (IP) injection at 0 or 40 mg/kg or oral gavage at 0, 300, 600 and 900 mg/kg (n = 4–5/group). Treatment resulted in dose-dependent increases in agmatine, but a similar reduction of putrescine, in the hippocampus. *Indicates significant treatment effects compared with saline at *p < 0.05, **p < 0.01 or ***p < 0.001.

In the hippocampus, there were no significant differences between groups in L-arginine (Fig. 4A), L-citrulline (Fig. 4B), L-ornithine (Fig. 4C), glutamine (Fig. 4D), glutamate (Fig. 4E), GABA (Fig. 4F), spermidine (Fig. 4I) and spermine (Fig. 4J) regardless of the delivery route and dose of agmatine. When agmatine levels were measured (Fig. 4G), IP injection resulted in a 284% increase in agmatine in the hippocampus (*P* = 0.03). Regarding oral agmatine treatment, one-way ANOVA revealed a highly significant difference between the four dose groups (*P* < 0.0009) with dose-dependent increases (2–7 folds) and higher agmatine levels at the 600 (*P* < 0.01) and 900 (*P* < 0.001) mg/kg doses. For putrescine, IP injection resulted in a trend of reduction in the hippocampus (*P* = 0.057). For the four oral treatment groups, there was a significant difference (*P* = 0.02) with lower levels of putrescine in the hippocampus (25–30% reduction) in the 300 and 600 mg/kg dose groups when compared to the saline group (all *P* < 0.05).

Based on the behavioural and neurochemical results of Experiment 1, the oral dose of agmatine at 300 mg/kg appeared to most closely resemble the behavioural and neurochemical effects of the IP injection of agmatine at 40 mg/kg. Hence agmatine (300 mg/kg, PO) was selected and used for the long-term treatment in Experiment 2.

### Experiment 2: Sub-chronic oral gavage agmatine treatment

#### Tolerability, body weight, food and water intake

**Figure 5:**
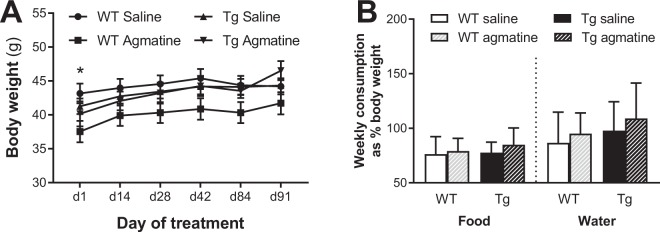
Effects of sub-chronic oral agmatine treatment on body weight and food and water consumption. Mean (±SEM) body weight over the treatment period (**A**) and weekly food and water consumption (**B**; presented as percentage of body weight) in wild-type (WT) and APP/PS1 (Tg) mice treated with saline or agmatine (300 mg/kg) via oral gavage over a period of 105 days (n = 13–16/group).

No mice died of treatment-related toxicity or adverse effects. All mice were healthy and survived to the end of the 105-day treatment period, indicating that repeated sub-chronic oral agmatine treatment was well tolerated in both WT and Tg mice. Animals’ body weighs were measured every two weeks on days 1, 14, 28, 42, 84 and 91 of treatment. Two-way repeated measures ANOVA revealed no significant group difference (*P* = 0.075), and all animals increased body weight over the course of the study (*P* < 0.0001; Fig. 5A). Bonferroni’s multiple comparison tests showed that WT mice receiving agmatine were significant lighter than WT saline mice on day 1 of treatment (*P* < 0.05), but this effect diminished during the treatment period. Food and water consumption were also measured during the first two months of treatment (normalised by body weight), and the mean data were used for analysis. There were no significant effects of genotype, treatment or their interaction for both measurements (Fig. 5B).

#### Behavioural results

**Figure 6:**
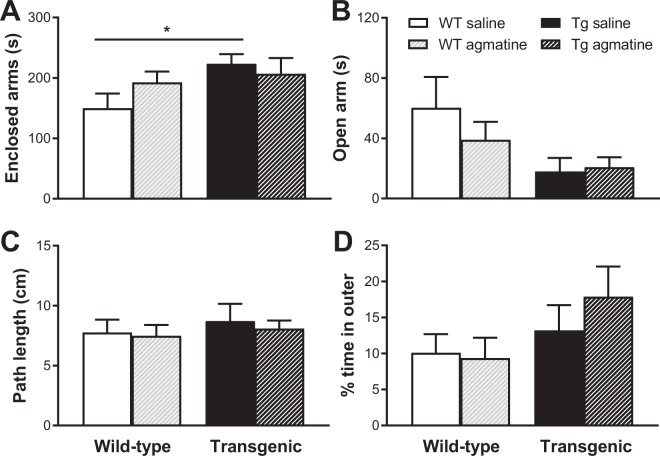
Behavioural effects of sub-chronic oral agmatine treatment. Mean (±SEM) time spent in enclosed (**A**) and open (**A**) arms of the elevated plus maze, and path length (**C**) and % time spent in the outer zone (**D**) of the open field apparatus in wild-type (WT) and APP/PS1 (Tg) mice treated with saline or agmatine (300 mg/kg) via oral gavage over a period of 105 days (n = 13–16/group).

All animals were tested in the elevated plus maze and open field on day 71 (approximately 24 h post-treatment). In the elevated plus maze, there were significant genotype effects in terms of the time spent in the enclosed and open arms (all *P* < 0.05), with saline treated Tg mice spending more time in the enclosed arms than saline treated WT mice (*P* < 0.05, Fig. 6A) and Tg mice spending less time in the open arms relative to WT mice regardless of treatment (*P* < 0.05). Animals in both genotype groups appeared to have more entries to the enclosed arms than the open arms, however with no genotype or treatment effect for each measurement (data not shown). There were no effects of genotype, treatment or their interaction in the path length travelled or the percentage of time spent in the outer zone of the open field (Fig. 6C,D).

#### Neurochemical results

**Figure 7:**
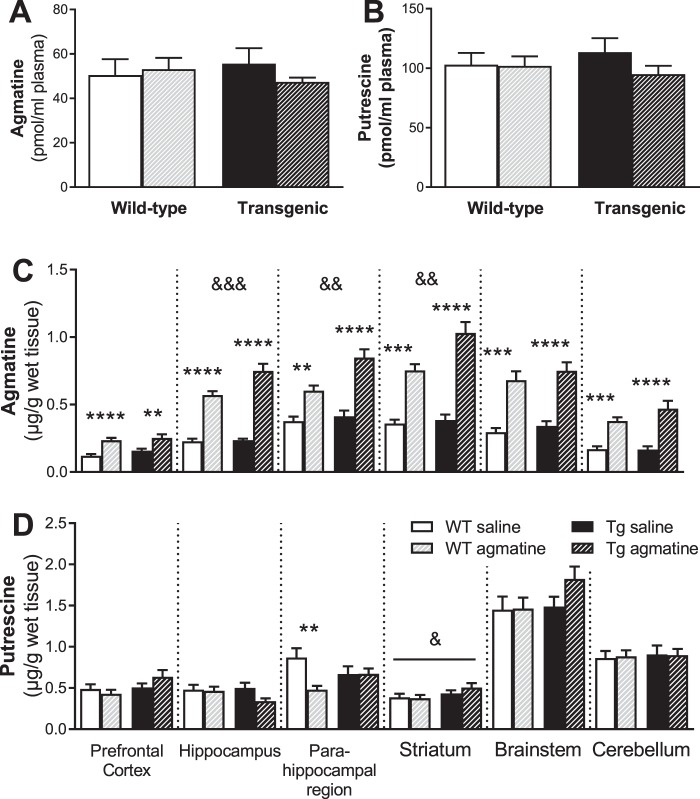
Effects of sub-chronic oral agmatine treatment on agmatine and putrescine in plasma and brain. Mean (±SEM) levels of agmatine (**A**,**C**) and putrescine (**B**,**D**) in plasma (**A**,**B**) and in the prefrontal cortex, hippocampus, parahippocampal region, striatum, brainstem and cerebellum (**C**,**D**) in wild-type (WT) and APP/PS1 (Tg) mice treated with saline or agmatine (300 mg/kg) (n = 13–16/group) by oral gavage on day 105 (24 hours after the final dosing on day 104). *Indicates significant treatment effects at **p < 0.01, ***p < 0.001 or ****p < 0.0001. ^&^Indicates significant genotype effects at ^&^*p* < 0.05, ^&&^*p* < 0.01 or ^&&&^*p* < 0.001.

Because agmatine treatment only significantly affected agmatine and putrescine levels in Experiment 1, these neurochemicals were the focus of Experiment 2. Regarding the blood profile, we found no significant effects of genotype, treatment and their interaction for plasma agmatine (Fig. 7A) and putrescine (Fig. 7B).

We further measured the agmatine levels in multiple brain regions. For the prefrontal cortex, two-way ANOVA revealed a significant treatment effect (*P* < 0.0001), but no genotype effect or genotype × treatment interaction, with markedly higher agmatine levels in the agmatine treated WT (*P* < 0.0001; 50% increase) and Tg (*P* < 0.01; 38% increase) mice relative to their corresponding genotype-matched saline controls. For the hippocampus, significant effects of genotype (*P* = 0.004), treatment (*P* < 0.0001) and their interaction (*P* = 0.008) were found, with higher agmatine levels in agmatine treated WT (60%) and Tg (70%) mice when compared to saline treated mice of the same genotype (all *P* < 0.0001) and in agmatine-treated Tg mice (25% increase) relative to agmatine-treated WT mice (*P* < 0.001). For the parahippocampal region, there were also significant effects of genotype (*P* = 0.002), treatment (*P* < 0.0001) and their interaction (*P* = 0.02), with higher agmatine levels in WT (*P* < 0.001; 38%) and Tg (*P* < 0.0001; 52%) mice received agmatine treatment relative to their corresponding genotype-matched saline controls and in agmatine-treated Tg mice (29% increase) when compared to agmatine-treated WT mice (*P* < 0.001). For the striatum, again there were significant effects of genotype (*P* = 0.004), treatment (*P* < 0.0001) and their interaction (*P* = 0.02), with higher agmatine levels in agmatine-treated WT (52%) and Tg (63%) mice relative to saline treated mice of the same genotype (all *P* < 0.0001) and in agmatine-treated Tg mice (27% increase) when compared to agmatine-treated WT mice (*P* < 0.001). For the brainstem and cerebellum, we found significant effects of treatment (brainstem: *P* < 0.0001, about 55% increase for both genotypes; cerebellum: *P* < 0.0001, 55% and 65% increases for WT and Tg mice respectively), but no genotype or interaction effects.

Regarding the brain putrescine profile, we found significant effects of treatment (*P* = 0.02) and genotype × treatment interaction (*P* = 0.02), but not genotype, in the parahippocampal region, with reduced putrescine levels in agmatine-treated WT mice when compared to saline-treated WT controls (*P* < 0.01). For the striatum, there was a significant effect of genotype (*P* < 0.05), but no genotype or genotype × treatment interaction, with higher levels in Tg mice relative to WT mice regardless of treatment. For the prefrontal cortex, hippocampus, brainstem and cerebellum regions, there were no other significant effects of genotype, treatment and their interaction.

#### Gross necroscopy, organ weights and histology

We further carried out post-mortem gross examinations of the heart, lung, liver, spleen, kidney, testis, gut and stomach, and found no apparent agmatine treatment-related changes in either WT or APP/PS1 mice. Their weights (except for the gut and stomach) are presented in Table 2 as a percentage of total body weight. For the heart, lung, liver, spleen, and testis, there were no significant effects of genotype, treatment and their interaction. However, Tg mice had significantly smaller kidney/body weight ratios relative to WT mice (*P* = 0.001), irrespective of treatment.

**Table 2:** Effects of sub-chronic agmatine treatment on organ weight.

Organ/body weight ratio	Brain	Heart	Kidney*	Liver	Spleen	Testicle
WT (Agm-0)	10.96 ± 0.37	3.97 ± 0.19	6.74 ± 0.46	47.11 ± 1.48	2.75 ± 0.14	2.30 ± 0.11
WT (Agm-300)	11.53 ± 0.38	4.21 ± 0.17	6.58 ± 0.22	43.27 ± 1.52	2.45 ± 0.14	2.29 ± 0.10
Tg (Agm-0)	10.63 ± 0.23	3.83 ± 0.09	5.82 ± 0.28	47.20 ± 1.55	2.69 ± 0.20	2.49 ± 0.13
Tg (Agm-300)	10.62 ± 0.29	3.77 ± 0.1	5.50 ± 0.22	47.93 ± 1.89	2.58 ± 0.19	2.22 ± 0.08

Mean (± SEM) organ (mg)/body (g) ratio of the whole brain, heart, kidney, liver, spleen or testicle in wildtype (WT) and APP/PS1 (Tg) mice with oral administration of agmatine sulfate at 0 (Agm-0) or 300 (Agm-300) mg/kg (n = 13–16/group). The organs were collected on day 105 (24 hours after the final dosing).

*Indicates a significant genotype difference in kidney (see section 3.2.4. for details).

An independent pathologist (Williams) carried out gross necroscopies, as well as the histological examinations on organ sections from all four experimental groups (n = 6–8/group). For the heart, spleen and testis, all were within normal limits, and normal spermatogenesis was seen in the testis. For the lungs, blood was seen in the airways and alveoli in most animals (most likely inhaled as an agonal event following euthanasia by decapitation). For the gut, examination of random pieces of bowel taken from a gut roll showed normal small and large bowel. Pinworms were seen in some animals. All stomachs were normal with no evidence of ulceration. For kidneys, animals in all four groups showed variable vacuoles in tubules within the cortex and occasionally in the medulla. Some animals had small areas of mononuclear cell infiltration in the region of the calyces, and the significance was not known. For the liver, animals in all four groups showed variable fatty change. Two Tg mice (irrespective of treatment) had a small hepatic adenoma. One agmatine-treated Tg mouse had a solid mass 12 × 7 × 4 mm attached to the liver, and histology showed this to be a hepatocellular carcinoma. Finally, one WT mouse treated with saline had extra tissue at the back of the neck, which was queried to be of possible developmental origin. This comprised muscle and bone (including an articular surface) were all normal.

## Discussion

Agmatine was discovered one hundred years ago. Due to its neuromodulatory, neuroprotective, anti-inflammatory, memory enhancing, anxiolytic and antidepressant properties, there has been a growing interest in clinical applications of agmatine^8,11,32,33^. Accordingly, the safety of agmatine supplementation has been paid a great attention. In humans, a randomized double-blind placebo-controlled trial demonstrated the safety and efficacy of oral agmatine sulfate (1,335–3,560 mg/day for up to 3 weeks) in lumbar disc-associated radiculopathy^34^. Moreover, a human case study reported no adverse effects of oral agmatine sulfate at a high daily dose of 2,670 mg (approximately 36–46 mg/kg) for a period of 5 years^22^. In rats, earlier studies demonstrated the safety of acute oral administration of agmatine sulfate up to 480 mg/kg and sub-chronic administration via drinking water at an estimated daily dose of 100 mg/kg for 90 days^18,19^. There were, however, significant reductions in water intake and body weight gain during the period of agmatine supplementation via drinking water perhaps due to the bitter taste of agmatine sulfate^19^. These findings raise a significant concern about the feasibility of using the drinking water delivery method for long-term agmatine supplementation. The present study, therefore, determined preclinical safety and tolerability of acute (Experiment 1) and sub-chronic (Experiment 2) agmatine treatment via oral gavage in mice. Moreover, we measured the levels of agmatine and arginine metabolites in the brain and plasma after the final dosing.

In Experiment 1, we first carried out a pilot dose-ranging study with a small sample size (4–5 animals/group), in which agmatine sulfate was given to 10-month WT mice once daily via oral gavage at the doses of 0, 300, 600 and 900 mg/kg for 7 days or by intraperitoneal injection at 0 and 40 mg/kg on days 6 and 7. There were no health complications noted for all of the animals that received treatment, and daily agmatine treatment up to 900 mg/kg for 1 week was well tolerated. While agmatine treatment at each oral dose did not affect animals’ body weight per se, all animals lost weight (about 1 g) irrespective of treatment over the course of a week (Table 1). Since no changes in body weight were noted over 100 days of gavaging in Experiment 2, the observed body weight changes in Experiment 1 are likely due to the acute stress associated with gavaging.

On day 6, there were no agmatine treatment-induced effects in the elevated plus maze and open field 30 min after dosing. There was an overall pattern of more time spent in the open arms in agmatine-treated animals regardless of the delivery route, which appears to be consistent with the well-documented anxiolytic effect of agmatine^28,35,36^. We also observed a clear pattern of shorter path lengths in animals received oral supplementation of agmatine relative to mice with saline treatment, perhaps due to agmatine induced inactivation of the NMDA receptor^2,8^.

The hippocampus and plasma were selected as the targets to understand the effects of agmatine treatment on L-arginine metabolism at peak concentrations (30 min after the final dosing on day 7). Oral delivery of agmatine resulted in dose-dependent 2–7 and nearly 500 folds increases in agmatine levels in the hippocampus and plasma respectively, with modest increases following IP injection of agmatine at 40 mg/kg. These results demonstrate that oral delivery of agmatine via gavage can rapidly pass the blood-brain barrier into the brain. It should be pointed out, however, that the animals were not flushed with saline prior to the brain tissue dissection, therefore the presence of agmatine in the cerebral capillaries may contribute to the agmatine tissue content in the hippocampus.

It is of interest to note that oral agmatine treatment resulted in decreased putrescine levels in the hippocampus regardless of dosage and delivery route, but increased putrescine levels in plasma in a dose-dependent manner, with no changes in L-arginine and other metabolites. Agmatine is known to be an alternative precursor for the putrescine biosynthesis through agmatinase and/or agmatinase-like protein (in rats), but can also induce antizyme to inhibit the activity of ornithine decarboxylase that converts L-ornithine to putrescine^7,8,37^. The differential pattern of agmatine-induced changes in putrescine in the brain and plasma demonstrates differential actions of exogenous agmatine on the central (brain) and peripheral organs, which merits future research to understand the underlying mechanisms and functional significance. The dose-independent reduction in putrescine in the hippocampus following the oral delivery of agmatine sulfate at 3 doses indicates that the affinity of agmatinase to exogenous agmatine might have reached saturation even at the lowest dose (300 mg/kg) in mice. Furthermore this finding suggests that previous studies reporting acute agmatine induced behavioural or neurochemical effects could be in part due to reduced putrescine levels, a notion not previously proposed due to underreporting of blood-brain neurochemistry.

We then carried out a sub-chronic oral gavage agmatine treatment (Experiment 2). The behavioural effects of long-term agmatine treatment were not significant when mice were tested in the elevated plus maze and open field 24 h after treatment on day 71. In the elevated plus maze, Tg mice spent more time in the enclosed arms relative to WT mice, a finding in opposition to previous studies^38,39^, and no significant genotype effects in open field behaviour are consistent with earlier research on APP/PS1 mice^40^. Interestingly, agmatine at 300 mg/kg did not affect animals’ performance in both tests, despite a trend of an anxiolytic effect of agmatine in Experiment 1, indicating the acute (peak concentration) effects of agmatine and putrescine on behavioural function.

In Experiment 2, gross necroscopy and histological examinations revealed that sub-chronic oral delivery of agmatine had no significant adverse effects on organ weights and tissue histology. Microscopic examination of the small and large intestines, as well as the stomach, confirmed that repeated sub-chronic bolus dosing with oral agmatine at 300 mg/kg over a period of 105 days was safe, with no noticeable ulceration in the stomach. Although a hepatocellular carcinoma was found in one of the agmatine-treated Tg mice, there was no evidence indicating abnormal cell growth or tumour formation due to repeated agmatine treatment. In fact, agmatine has been reported to exert anti-proliferative effects on tumour growth^9,20,41–43^. Moreover, it is important to note that sub-chronic oral delivery of agmatine did not affect the organ/body weight ratios for the heart, lung, liver, spleen, and testis. While smaller kidney/body weight ratios were found in Tg mice relative to WT mice irrespective of treatment, there were no marked gross or microscopic changes in kidneys between two genotype groups.

In Experiment 2, the brain tissue (prefrontal cortex, hippocampus, parahippocampal region, striatum, brainstem and cerebellum) and plasma were collected 24 h after the final dosing. Agmatine and putrescine levels were then determined, since they were the only neurochemical variables changed in Experiment 1. We found significantly increased agmatine levels in all six brain regions, but not plasma, in agmatine treated mice regardless of genotype, indicating the accumulation of exogenous agmatine in the brain tissue. Interestingly, Tg mice with agmatine treatment had significantly higher levels of agmatine in the hippocampus, parahippocampal region and striatum relative to agmatine-treated WT mice, suggesting that APP/PS1 mice at 13 months of age may have increased permeability for agmatine due to impaired BBB^44^ or increased storage capacity in the brain. Under physiological conditions, exogenous agmatine has limited ability to cross the BBB^21^. There are, however, long lasting increases in BBB permeability under ischemic conditions^45^. It has been shown that the Tg brain may be under ischemic conditions^46^, which may account for genotype-specific increases of agmatine in the brain of APP/PS1 mice. More research is required to understand the exact mechanisms underlying the observed changes, but increased agmatine levels in the amyloid burdened brain may be advantageous.

It’s of interest to note that there were no significant effects of agmatine sulfate treatment on the putrescine levels in plasma when measured 24 hours after dosing (Experiment 2). This finding suggests that increased plasma putrescine levels following agmatine treatment at 30 min post-treatment (Experiment 1) relate to the peak levels of agmatine and the changes disappear over time. In Experiment 1, oral agmatine sulfate at 300 mg/kg led to a significant reduction in putrescine in the hippocampus 30 min post-treatment (Fig. 3H). In Experiment 2, however, there was no such reduction in putrescine in the hippocampus, as well as the frontal cortex, striatum, brainstem or cerebellum, although reduced putrescine levels were seen in WT (but not Tg) mice with agmatine treatment. We also observed a significant genotype effect in the striatum with higher levels of putrescine in Tg mice irrespective of treatment, which merits future investigation. Taking agmatine and putrescine data from both experiments together, it appears that exogenous agmatine is safely transported to and stored in the brain after initial high concentrations following oral administration, and its effects on putrescine seem to be transient. Despite the high doses of agmatine used here to avoid potential floor effects due to reportedly low BBB permeability, rapid clearance and high first pass metabolism properties^20,21^, it is worth considering that treatment with lower agmatine doses that previously have shown behavioural efficacy and neurochemical changes^8,10^ should in theory also promote elevated levels of agmatine in the hippocampus of mice. Thereby postulating what dose-response and therefore minimal increase in brain agmatine levels is advantageous? Further studies that report agmatine-induced changes perhaps might also consider reporting the biological levels of agmatine (and putrescine) required for these effects.

In conclusion, the present study demonstrates that oral administration of agmatine sulfate via gavage at doses up to 900 mg/kg (once daily for 7 days) or at 300 mg/kg (once daily for 105 days) is safe for adult male mice. Moreover, oral delivery of agmatine via gavage rapidly crosses the BBB and accumulates in the brain following repeated dosing, with higher concentrations in some brain regions of APP/PS1 mice with chronic amyloid deposition relative to that of WT mice. These findings raise an important issue in the area of the translational research for agmatine, as the individual dosing needs to be carefully considered based on the situation, although agmatine is safe in general. Another interesting finding is that exogenous agmatine seems to have differential effects on polyamines in the brain and blood as evidenced by the putrescine results in Experiment 1. Since physiological levels of polyamines are essential in maintaining normal cellular function, the effects of exogenous agmatine on polyamines and other bioactive metabolites of L-arginine should be considered and reported.

## Materials and Methods

### Animals

Male B6C3-Tg(APPswe,PSEN1ΔE9)85Dbo/Mmjax (stock number 004462) and B6C3 wild-type (WT) females (Jackson Laboratory) were crossed resulting in hemizygote APPswe/PS1ΔE9 transgenic (Tg) and WT litter-mates. Nine to 10 month old mice weighing between 37–42 gm were individually housed (13 × 15 × 38 cm^3^), maintained on a 12-h light/dark cycle (lights on at 8 AM) and provided *ad libitium* access to food and water, with experimental procedures carried out during the light phase. Experiments were first approved then conducted in accordance with the regulations of University of Otago Ethics in the Care and Use of Laboratory Animals Committee (AEC protocol number 23/11). Every attempt was made to reduce the numbers of animals used and to refine techniques to reduce suffering.

### Drug treatment

Agmatine sulphate (Sigma, Sydney; A7127) was dissolved in saline twice per week and stored in the fridge. Our previous work demonstrated the stability of agmatine in saline or water at room temperature and 4 °C for at least 4 and 7 days respectively^47^. In Experiment 1 (short-term dose-ranging study), twenty-seven 10-month WT mice were randomly divided into 6 groups: saline (1 ml/kg, IP, *n* = 4), agmatine (40 mg/kg, IP, *n* = 4), saline (4 ml/kg, p.o., *n* = 4), agmatine (300 mg/kg, p.o., *n* = 5), agmatine (600 mg/kg, p.o., *n* = 5) and agmatine (900 mg/kg, p.o., *n* = 5). The dose of agmatine (40 mg/kg, IP) was selected based on our previous research^23,28–30^, whereas a dose range of oral agmatine at 0–900 mg/kg was selected based on earlier research^18^. All animals were pre-handled for 2 min daily for 5 days, then treated for two days with 4 ml/kg saline p.o. On the following 7 days, animals were treated once daily with their respective drug regimens except for the IP treated groups who received saline p.o. once daily for 5 days and were then injected with saline or agmatine 30 min prior to behavioural testing on day 6 and 30 min prior to sacrifice day 7 (Fig. 1). The 30 min timepoint was selected to investigate the peak concentration effects of agmatine as part of an acute treatment regime based on a pilot study.

In Experiment 2 (sub-chronic agmatine treatment), mice were treated with saline or agmatine at 300 mg/kg, the dose that most closely resembled the behavioural and neurochemical effects of the IP injection of agmatine (40 mg/kg) in Experiment 1. Fifty-seven mice (27 Tg and 30 WT), 9 months of age, were randomly divided into 4 groups: WT saline (4 ml/kg, p.o., *n* = 14), WT agmatine (300 mg/kg, p.o., *n* = 16), Tg saline (4 ml/kg, p.o., *n* = 14) or Tg agmatine (300 mg/kg, p.o., *n* = 13). Animals were treated once daily by oral gavage 5 days per week up to 8 weeks, then every day up to day 104 (Fig. 1). The 24 hour post-treatment timepoint for both behavioural testing and tissue collection detailed below was selected to avoid the peak agmatine effects on animals’ behaviour and brain and blood neurochemistry, hence allowing for the assessment of possible accumulation of agmatine in the tissues over a long period of treatment. For both experiments, animals were gavaged with a volume of 4 ml/kg.

### Behavioural procedures

The elevated plus maze had four arms (65 × 5 cm), two open arms surrounded by 1 cm clear Plexiglas and two closed arms surrounded by 15 cm high white Plexiglas. The central area of the maze measured 5 × 5 cm. The open field apparatus was a 40 × 40 cm white Plexiglas box with identical 20 cm high walls. Animals were tested for 5 min in the elevated plus maze then the open field, 30 min post-gavage on day 6 (Experiment 1) or 24 hr post treatment on day 71 (Experiment 2) (Fig. 1). For the elevated plus maze, the duration of time spent in and the number of entries to the enclosed and open arms were analysed. For the open field, the path length animals travelled and the percentage of time spent in the outer zone (10 cm from the wall) were analysed offline using HVS 2020 (HVS Image Software Ltd, Bicester, England).

### Blood and brain tissue preparation

Animals were decapitated without anaesthesia 30 min (Experiment 1) or 24 h (Experiment 2) following the final agmatine treatment on day 7 and day 105, respectively (Fig. 1). Sample collection, preparation procedures and neurochemical assays were identical for both experiments, but conducted independently, and as detailed previously^26^. Briefly trunk blood was collected in an EDTA coated tube and centrifuged at 2,000 *g* for 10 min at 4 °C (Eppendorf 5810) to obtain plasma. Ice-cold ethanol/methanol (50/50; v/v) was added to the plasma samples at a 4:1 ratio, and the mixture was centrifuged at 12,000 g for 10 min at 4 °C^26^. The supernatant from each tube was stored at −80 °C until high performance liquid chromatography (HPLC) and liquid chromatography/mass spectrometric (LC/MS) assays.

The brain from each animal was rapidly removed and transferred to cold saline for at least 45 s. The whole hippocampus (for both experiments) and the prefrontal cortex, parahippocampal region (containing the entorhinal, perirhinal and postrhinal cortices), striatum, brainstem and cerebellum (Experiment 2 only) were then dissected on ice as described previously^26^. Brain tissue samples were weighed, homogenized in ice-cold 10% perchloric acid (~50 mg wet weight per mL) and centrifuged at 10,000 *g* for 10 min at 4 °C to precipitate protein. The perchloric acid extracts (supernatants) were then stored at −80 °C until the HPLC and LC/MS assays.

### Neurochemical procedures

The brain and plasma levels of amino acids (L-arginine, L-citrulline, L-ornithine, glutamate and GABA) and the brain polyamines spermidine and spermine were quantified by HPLC, while the brain and plasma agmatine and putrescine levels and plasma spermidine and spermine levels were measured by LC/MS^24–27^. For each experiment, plasma or brain samples from all experimental groups were assayed at the same time, in duplicate, in a counterbalanced manner. The concentrations of L-arginine and its nine downstream metabolites in brain tissue or plasma were calculated with reference to the peak area of external standards. Values were expressed as μg/g or mg/g wet brain tissue, and pmol/ml or nmol/ml of plasma^26^.

### Necroscopy and histological examination

In Experiment 2, a gross necroscopy was conducted for each animal following removal of the head. The major organs (liver, kidney, spleen, stomach, small or large intestine, lung, heart and testicles) were collected, weighed (except for the gastrointestinal tract, which was flushed with saline and rolled around a toothpick into a “gut roll”) and immersed in 10% formalin, before histological examination by the University of Otago Histology Unit. A representative sample of each tissue was blocked, sliced and stained using haematoxylin and eosin. Six to eight animals from each of the four groups were randomly selected, and organ sections were independently examined by a pathologist.

### Statistical analysis

For Experiment 1, the data obtained from the IP and oral gavage groups were analysed separately. Body weight was analysed by two-way analysis of variance (ANOVA), whereas behavioural and neurochemical data was analysed by non-parametric Mann Whitney or Kruskal-Wallis tests followed by Dunn’s multiple comparison tests. For Experiment 2, body weight, behavioural and neurochemical data were analysed by two-way ANOVA followed by Tukey post-hoc tests. All calculations were performed using Graphpad Prism and significance was set to p < 0.05 for all comparisons.

## Data Availability

All data generated or analysed during this study are included in this published article.
